# Hydrogen-Rich Saline Promotes the Recovery of Renal Function after Ischemia/Reperfusion Injury in Rats via Anti-apoptosis and Anti-inflammation

**DOI:** 10.3389/fphar.2016.00106

**Published:** 2016-04-22

**Authors:** Jie Li, Zhijian Hong, Hong Liu, Jihong Zhou, Lei Cui, Siming Yuan, Xianghua Chu, Pan Yu

**Affiliations:** ^1^Department of Burn and Plastic Surgery, Jinling HospitalNanjing, China; ^2^Department of Nephrology, Yongchuan Hospital of Chongqing Medical UniversityChongqing, China; ^3^Department of Nephrology, Hospital of Traditional Chinese MedicineChongqing, China; ^4^Department of Pharmacy, The Affiliated Hospital of Qingdao UniversityQingdao, China

**Keywords:** hydrogen, recovery of function, ischemia/reperfusion, kidney, apoptosis

## Abstract

**Purpose:** Hydrogen is a proven novel antioxidant that selectively reduces hydroxyl radicals. In this study, we investigated the effects of hydrogen-rich saline solution on the prevention of renal injury induced by ischemia/reperfusion (I/R) and on renal function recovery.

**Methods:** A rat model of renal I/R injury was induced by 45 min occlusion of the left renal pedicle, followed by 108 h reperfusion. The right kidney was surgically removed. Then, 0.9% NaCl solution (1 ml/kg) or hydrogen-rich saline solution (HRSS; 1 ml/kg) was injected into the abdominal cavity at 4 h intervals. We assessed the influence of HRSS or control saline solution on the recovery of renal function after I/R injury. Kidney tissues were taken at different time points (24, 36, 48, 72, and 108 h after reperfusion) and frozen (-80°C). Kidney cell apoptosis was evaluated using terminal deoxynucleotidyl transferase dUTP nick end labeling (TUNEL)-positive staining. Additionally, the apoptotic factors (Bcl-2, Bax, caspase-3, caspase-9, and caspase-8) and the pro-inflammatory cytokines (IL-6 and TNF-α) were measured in the kidney tissues. Finally, serum blood urea nitrogen (BUN) and creatinine (Cr) levels were measured.

**Results:** Histological analyses revealed a marked reduction of interstitial congestion, edema and hemorrhage in renal tissue after HRSS treatment compared to saline treatment. After I/R injury, BUN, Cr, Bcl-2, caspase-3, caspase-9, caspase-8, IL-6, and TNF-α were all significantly increased, while Bax expression was decreased. HRSS remarkably reversed these changes. Moreover, BUN and Cr decreased more rapidly in the rats treated with HRSS compared to the rats treated with control saline solution.

**Conclusions:** HRSS showed a protective effect in the prevention of renal injury and could promote renal function recovery after I/R injury in rats. HRSS might partially exert its role through an anti-apoptotic and anti-inflammatory action in kidney cells.

## Introduction

Renal ischemia-reperfusion (I/R), an important cause of acute kidney injury, is unavoidable during various types of operations, such as renal transplantation, surgical revascularization of the renal artery, partial nephrectomy and treatment of suprarenal aortic aneurysms (Perico et al., 2004; Sharples et al., 2005; Habib et al., 2015; Stangenberg et al., 2015). The mechanisms responsible for the pathogenesis of renal damage remain largely unknown, although reactive oxygen species (ROS) and apoptosis seem to be involved (Sun et al., 2016). Experimental and clinical studies have demonstrated the pivotal role of oxidative stress that is associated with ROS overproduction. Oxidative stress produces a large number of inflammatory factors, may cause lipid peroxidation compromising the integrity of cellular membranes, and promoting renal injury and cell apoptosis. In addition, ROS, including the superoxide anion (O_2_^-^), hydrogen peroxide (H_2_O_2_) and hydroxyl radicals (ΔOH), are believed to play an important role in secondary injury (Fukui et al., 2002; Sheu et al., 2006; Melcher and Moerschbacher, 2016). At high concentrations, ROS cause cytotoxicity by damaging proteins and nucleic acids, which leads to kidney cell apoptosis. Consequently, anti-apoptotic and anti-inflammatory treatments are being developed for kidney injury treatment.

Recently, it was demonstrated that molecular hydrogen (H_2_) could selectively reduce cytotoxic ROS and reactive nitrogen species, such as ΔOH and ONOO^-^ *in vitro*, and could exert therapeutic antioxidant and anti-inflammatory activity in many animal models (Fukuda et al., 2007; Ohsawa et al., 2007). H_2_ is present in almost every chemical compound and everywhere in nature. Its mild reductive reactivity minimizes its disturbance on metabolic oxidation-reduction and ROS-related cell signaling, avoiding serious side effects in medical procedures (Hayashida et al., 2008). In addition, H_2_ is advantageous over many other antioxidants because it can penetrate biomembranes as a gas. Its diffusion to cellular organelles helps to effectively target intracellular inflammatory factors. Previous studies showed that the inhalation of H_2_ exerts protective effects on brain, liver, heart, and intestinal I/R injuries (Bali et al., 2015; Malik and Ferguson, 2015; Du et al., 2016; Nagpure and Bian, 2016). Moreover, H_2_ acts as an anti-inflammatory agent in acute pancreatitis, colon inflammation and liver inflammation (Cheng et al., 2014; Yemm et al., 2015; Han et al., 2016).

However, H_2_ gas inhalation as a clinical application is not convenient and may be dangerous because of its high flammability. Compared with hydrogen gas, H_2_ saturated saline solution (i.e., a hydrogen-rich saline solution, HRSS) is safe and easy to administer. HRSS has also been implicated in attenuating I/R-induced renal injury (Shingu et al., 2010; Wang et al., 2011, 2014). Some studies have also shown that H_2_ can protect the kidneys from drug injuries (Matsushita et al., 2011; Katakura et al., 2012). It remains unknown, however, whether HRSS can facilitate renal function recovery after I/R injury in rats. Therefore, the present study aims to investigate HRSS therapeutic effects on renal I/R injury after 108 h reperfusion. In addition, we further analyzed the hydrogen mechanism of action against renal I/R injury.

## Materials and Methods

### Hydrogen-Rich Saline Solution Preparation

Hydrogen-rich saline solution was prepared as previously described (Cai et al., 2009). Briefly, hydrogen was dissolved in saline solution for 6 h under high pressure (0.4 MPa) using an apparatus manufactured in our department. The saturated hydrogen saline solution was stored under atmospheric pressure at 4°C in an aluminum bag with no dead volume. HRSS was sterilized by gamma radiation. To ensure that a concentration of 0.6 mmol/L was maintained, HRSS was freshly prepared every week. The hydrogen content of HRSS was confirmed using the gas chromatography method described by Ohsawa et al. (2007).

### Animals

Sprague Dawley (SD) rats (250–300 g) were kept in standard cages under a 12-h light/dark cycle. All experimental procedures were approved by the Institutional Animal Care and Use Committee of Jinling Hospital (Nanjing, China).

### Experimental Protocols

Animals were divided into four groups: (1) sham-operated rats receiving vehicle physiological saline solution treatment (control group, *n* = 30); (2) I/R rats receiving vehicle physiological saline solution treatment (I/R group, *n* = 30); (3) I/R rats receiving HRSS treatment at 4 h intervals for a total of 108 h (long-term treatment group, *n* = 30); (4) I/R rats receiving HRSS treatment for 24 h and then vehicle physiological saline solution treatment for 84 h (short-term treatment group, *n* = 30). In the control group, rats received a sham operation, followed by a continuous intraperitoneal administration of 0.9% NaCl solution (1 ml/kg). In the I/R group, the right kidney was surgically removed, and the left renal artery was occluded. This operation was followed by intraperitoneal administration of 0.9% NaCl solution (1 ml/kg) at 4 h intervals. Left renal artery occlusion was performed for 45 min under pentobarbital anesthesia. In the HRSS-I/R groups, the right kidney was surgically removed, and the left renal artery was occluded (Shingu et al., 2010; Wang et al., 2011). This was followed by intraperitoneal administration of HRSS (1 ml/kg) immediately after reperfusion at 4 h intervals. Occlusion of the left renal artery was performed as described above. Rats received saline solution or HRSS immediately after reperfusion at 4 h intervals for a total of 108 h. The animals were anesthetized by intraperitoneal injection of pentobarbital (Biomics Laibo, Beijing; 0.7 mg/kg) before any surgical treatments.

### Analysis of Renal Function

Blood urea nitrogen and Cr were measured to evaluate renal function. Samples were analyzed using commercial kits (Sigma, St. Louis, MO, USA) and a COBAS Mira chemical analyzer (Roche, Basel, Switzerland).

### Histological Analyses

Renal samples were embedded in paraffin, cut into 4 μm sections and stained with hematoxylin and eosin (H&E). Samples evaluation was performed under light microscopy by experienced technicians blinded to the groups. These expert observers made all assessments. To assess tubular injury, the percentages of tubules in the outer medulla and corticomedullary junction with tubular cell necrosis, cytoplasmic vacuole formation, hemorrhage and tubular dilatation were measured. Semi-quantitative injury scores ranged from 0 to 5 (0: normal kidney; 1: 0–10% injury; 2: 11–25% injury; 3: 26–45% injury; 4: 46–75% injury; 5: 76–100% injury).

### *In Situ* Apoptosis Assay

Kidneys were perfused with PBS (50 ml) using the transcardiac approach, followed by 4% phosphate-buffered formalin. Perfusion-fixed kidney tissues were further fixed overnight in a solution of 4% paraformaldehyde in PBS, embedded in paraffin and cut into 4 μm serial sections. Terminal deoxynucleotidyl transferase dUTP nick end labeling (TUNEL) staining was performed on paraffin embedded sections by using an *in situ* cell death detection kit (Roche, Indianapolis, USA). According to standard protocols, the sections were dewaxed and rehydrated by heating the slides at 60°C. Then, these sections were incubated in a proteinase K working solution (20 mg/ml) for 15 min at room temperature. The slides were rinsed three times with PBS before the incubation in the TUNEL reaction mixture for 1 h at 37°C. The area around the sample was dried by filter paper, and Converter-AP was added to the samples for 1 h at 37°C. After rinsing with PBS (5 min, three times), sections were stained with nitroblue tetrazolium (NBT) and 5-bromo-4-chloro-3-indolylphosphate (BCIP) until they became dark. Four slide fields were randomly examined using a defined rectangular field area at 100× magnification. One hundred cells were counted in each field. Data are shown as percentage of TUNEL-positive cells of the total cell nuclei per field.

### Immunohistochemistry

Bax and Bcl-2 imunohistochemical evaluation was carried out as previously described (Nakamura et al., 2007). Five μm tissue sections were deparaffinized, rehydrated in gradient alcohols and processed using the streptavidin immunoperoxidase method. In brief, sections were submitted to antigen retrieval by microwave oven treatment for 10 min in 0.01 mol/L citrate buffer (pH 6.0). Slides were then incubated in 10% normal serum for 30 min, followed by an overnight incubation at 4°C with the appropriately diluted primary antibody. Bax and Bcl-2 antibodies (Beyotime Inc., China) were used at 1:100 dilution. Next, samples were incubated with biotinylated anti-mice or anti-rabbit immunoglobulins for 15 min at 37°C. Then, they were incubated with streptavidin peroxidase complexes for 15 min at 37°C. Four slide fields were randomly examined using a defined rectangular field area at 400× magnification. Bax and Bcl-2 positive cells were counted in each field. Data are shown as the number of Bax and Bcl-2 positive cells per field.

### Cytokine Measurement in Kidney Tissues

Kidney tissues were collected and washed with normal saline solution. Then, they were immediately homogenized in 1 ml of cold normal saline solution (4°C) on ice. The homogenates were centrifuged at 3,000 × *g* at 4°C for 15 min. Caspase-3, caspase-9, and caspase-8 activities were evaluated using a Fluorometric Assay Kit (BIOVISION Research Products, Mountain View, CA, USA), according to the manufacturer’s instructions. IL-6 and TNF-α were measured with a commercial ELISA kit, according to the manufacturer’s instructions (Dakawe, Shenzhen, China).

### Statistical Analyses

Data are expressed as mean ± standard deviation (SD). Each experiment was repeated three times, and data represent the means from at least three parallel samples. SPSS version 11.0 (SPSS Inc., Chicago, USA) was used for all statistical analyses. Analysis of variance (ANOVA) and Student’s *t*-test were used to determine the differences among and between groups, respectively. *P*-values of less than 0.05 were considered statistically significant.

## Results

### Hydrogen Exerted a Protective Effect on I/R-Induced Renal Injury

**FIGURE 1:**
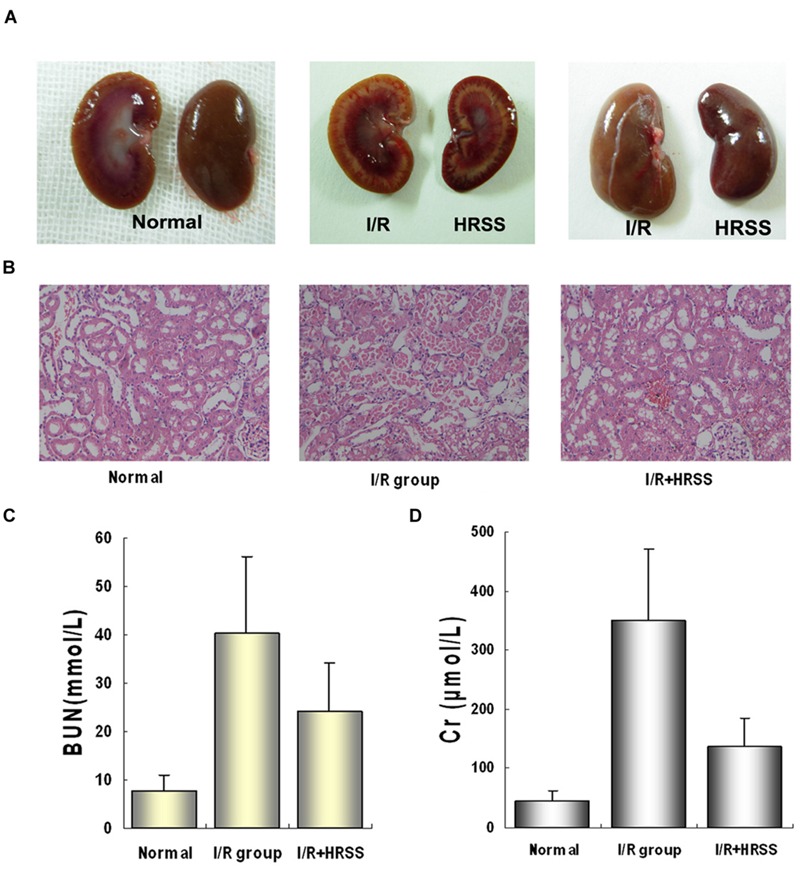
**Alterations in renal morphology and function before and after renal I/R injury with and without hydrogen-rich saline solution (HRSS) treatment. (A)** Kidneys gross structure. **(B)** Histological examination by H&E staining (×100). **(C)** Renal function at 24 h after I/R injury with and without HRSS treatment. **(D)** Creatine (Cr) and **(C)** blood urea nitrogen (BUN) were significantly reduced in HRSS-treated kidneys (*P* < 0.01).

In a preliminary study of hydrogen’s protective effect in kidneys, we analyzed kidney morphology and function at 24 h after I/R injury, and we found similar results to previous studies (Shingu et al., 2010; Wang et al., 2011, 2014). As shown in **Figure 1**, HRSS significantly reduced renal I/R injury. After HRSS treatment, renal tubule denaturation and edema were significantly attenuated (**Figure 1A**). Histology results in the normal kidney show that the renal glomerulus and tubules structures were clear without hemorrhage in the renal tubule, without loss of epithelial cells. In contrast, renal tubules in the I/R injured kidney showed evident edema, an almost complete loss of the epithelium and infiltration of red blood cells. The HRSS-treated kidney showed a partial loss of the epithelium in the renal tubules, and the number of red blood cells was significantly decreased, compared to the I/R injured kidney (**Figure 1B**). Additionally, serum Cr and BUN levels of the rats in the HRSS treatment group were significantly reduced when compared to the control group (*P* < 0.01; **Figure 1C**).

Additionally, we detected renal cell apoptosis by TUNEL immunostaining. As shown in **Figures 4A,B**, TUNEL-positive renal cells were significantly reduced at 24 h after I/R injury in the short-term and long-term treatment groups when compared to the I/R group. However, in the short-term treatment group, renal cell apoptosis still occurred after discontinuation of HRSS treatment, while it remained significantly reduced in the long-term treatment group.

In order to further verify HRSS therapeutic effect on renal I/R injury, we analyzed kidney morphology by H&E staining after I/R injury. At 24 h after I/R injury, renal tubular necrosis was significantly reduced in the short-term and long-term treatment groups. Renal hemorrhage and edema were also significantly attenuated, compared to the I/R group. Continuous HRSS treatment accelerated the recovery of the renal tubules function, when compared with short-term treatment. Histology scores for renal injury showed similar results (**Figure 3**).

### Hydrogen Could Promote the Recovery of Renal Function

**FIGURE 2:**
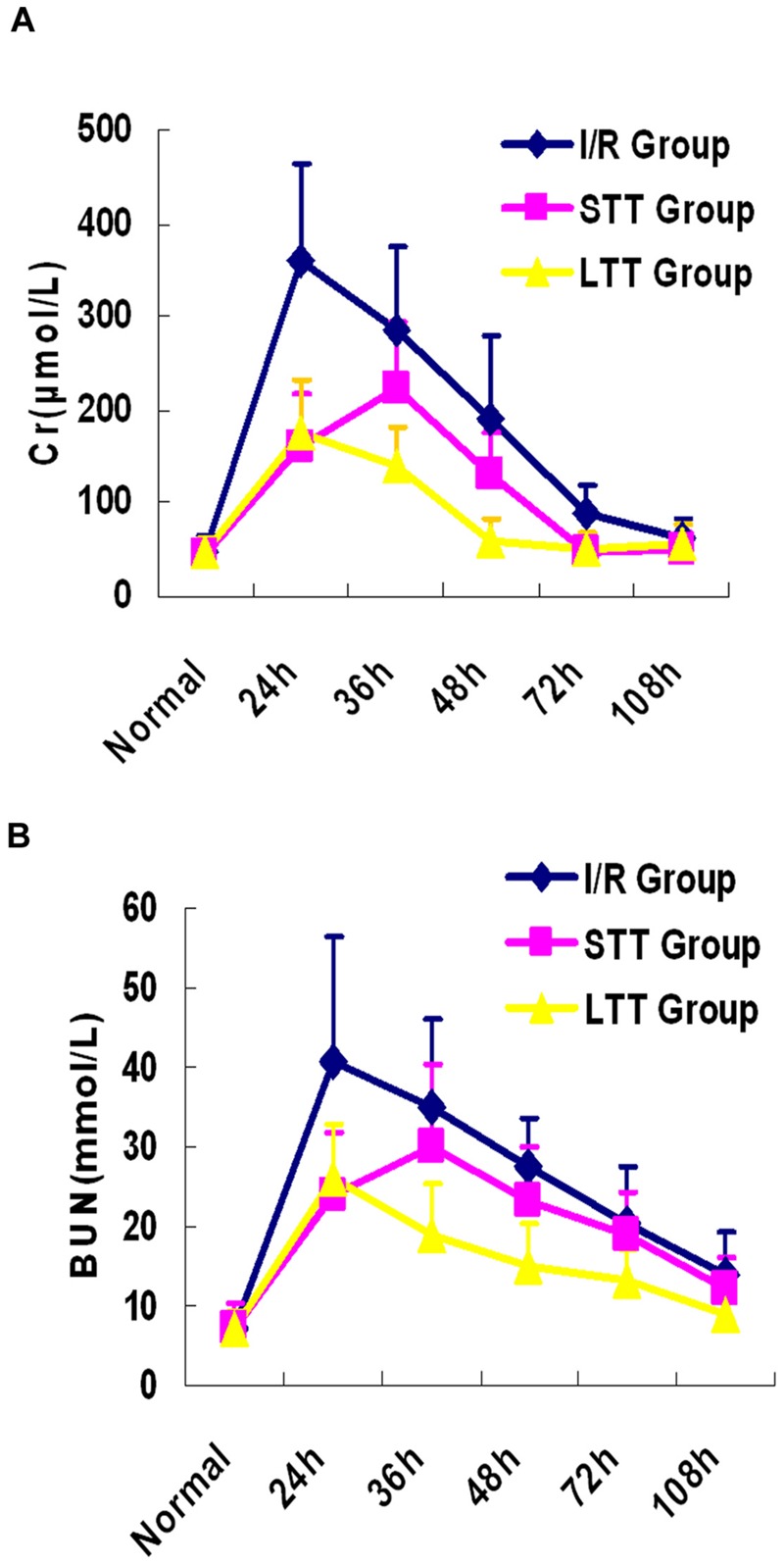
**Long-term HRSS treatment attenuated renal I/R injury.** Short-term HRSS treatment (STT) after renal I/R injury reduced renal injury, but renal function deteriorated once the treatment was stopped. In contrast, long-term HRSS treatment (LTT) markedly reduced renal injury and accelerated renal function recovery. **(A)** Blood creatinine. **(B)** Blood urea nitrogen.

Previous studies on hydrogen and I/R injury mainly focus on the ability of hydrogen on inhibiting ROS to reduce renal I/R injury. It has been well established, however, that the oxidation reaction after I/R injury is a lengthy process and can cause sustained renal injury. Therefore, we speculated that continuous HRSS treatment could promote renal function recovery. To test this hypothesis, we monitored renal function at different time points after I/R injury. When compared with the I/R group, the long-term and short-term treatment groups showed significantly reduced renal function impairment, and the renal function was rapidly recovered. However, compared with the short-term treatment group, renal function recovery in the long-term treatment group was faster. In addition, we also unexpectedly found that serum Cr and BUN increased in the short-term treatment group after discontinuation of HRSS treatment (**Figure 2**). These results indicated that after renal I/R injury, continuous HRSS treatment exerted a significantly enhanced therapeutic effect and promoted more rapid renal function recovery than short-term HRSS treatment.

**FIGURE 3:**
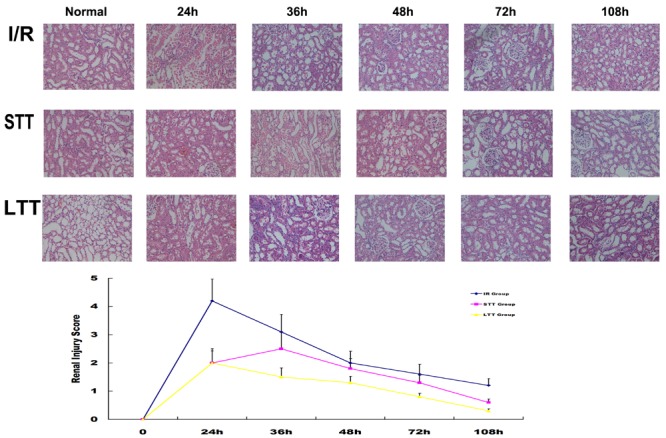
**Long-term HRSS treatment prevented delayed injury after renal I/R injury.** In the STT group, delayed renal injury, such as renal edema, epithelial cell loss and increased red blood cells in the renal tubule, occurred once the treatment stopped and were suppressed by long-term HRSS treatment. Histological changes observed after renal I/R injury included tubular cell necrosis, cytoplasmic vacuole formation, hemorrhage and tubular dilatation. Injury scores are expressed as mean ± SD. Semi-quantitative injury scores ranged from 0 to 5 (0: normal kidney; 1: 0–10% injury; 2: 11–25% injury; 3: 26–45% injury; 4: 46–75% injury; 5: 76–100% injury).

### Effect of HRSS on Pro-inflammatory Cytokines and Apoptotic Factors’ Levels

**FIGURE 4:**
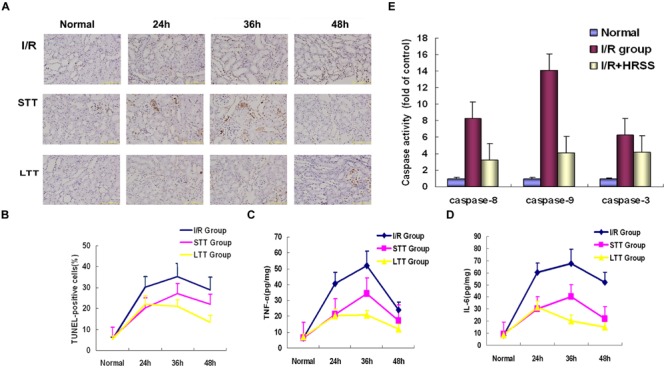
**Hydrogen inhibited renal cell apoptosis after renal I/R injury.** Apoptosis in I/R kidneys receiving long-term or short-term HRSS-treatment **(A,B;** ×100), TNF-α **(C)** and IL-6 **(D)** expression in the STT and LTT groups were reduced (*P* < 0.05). **(E)** Caspase-9, caspase-8, and caspase-3 expressions were markedly decreased in the kidneys of rats in the LTT group (*P* < 0.01).

In order to determine the effect of HRSS treatment on inflammatory cytokine levels in kidney tissue, we measured TNF-α and IL-6 expression by ELISA. As shown in **Figures 4C,D**, inflammatory cytokines levels in the short-term and long-term treatment groups were significantly reduced at 24 h after I/R injury, when compared to the I/R group. However, after discontinuation of HRSS treatment, the inflammatory cytokine levels in the kidney tissues of the short-term treatment group were increased, while in the long-term treatment group these levels continuously decreased.

**FIGURE 5:**
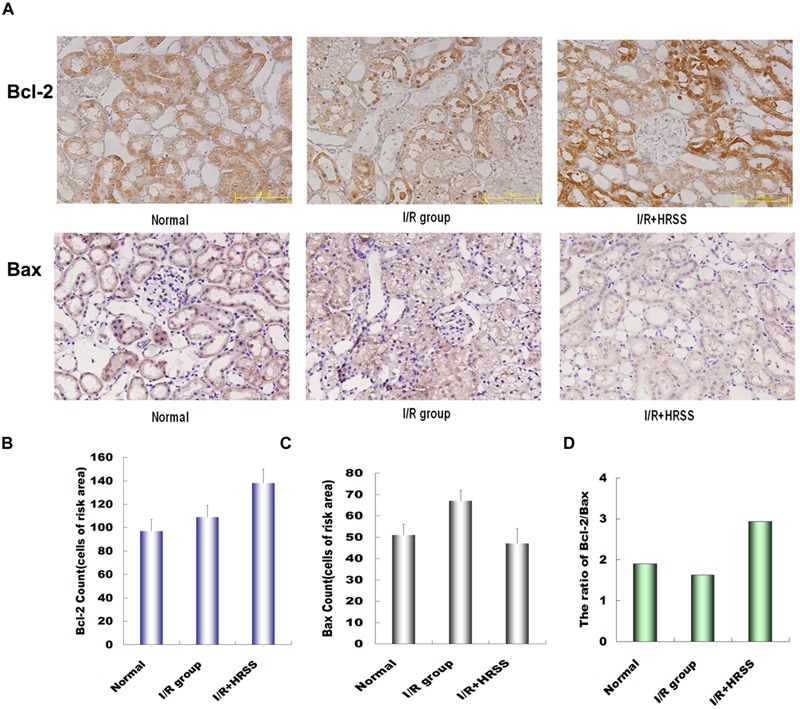
**(A)** Hydrogen modulated Bcl-2 and Bax expression in renal tissue after I/R injury. **(B)** Bcl-2 expression was upregulated, while **(C)** Bax expression was downregulated in renal tissue (*P* > 0.05). Although the change in Bax expression was not significant, **(D)** Bcl-2/Bax ratio was significantly altered (*P* < 0.05).

To further study the mechanism by which HRSS reduces renal cell apoptosis, Bcl-2 and Bax renal expressions were analyzed at 24 h after I/R injury by immunohistochemistry (**Figure 5A**). Bcl-2 expression in the HRSS treatment group was increased compared to the I/R group, while Bax expression was slightly decreased. However, these differences between the two groups were not statistically significant (*P* > 0.05; **Figures 5B,C**). We also compared the Bcl-2/Bax ratios in the I/R and hydrogen treatment groups, and we found that this ratio was significantly increased in the HRSS treatment group (*P* < 0.05; **Figure 5D**).

Because activation of caspase-3, caspase-8, and caspase-9 plays an important role in apoptosis induction, we analyzed their expression in kidney tissue. As shown in **Figure 4E**, caspase-3, caspase-8, and caspase-9 levels in the short-term and long-term treatment groups were significantly reduced compared to the I/R group (*P* < 0.01; **Figure 4E**).

## Discussion

This study showed HRSS ability to promote renal function recovery after I/R injury in rats by inhibiting the inflammatory TNF-α /IL-6 pathway, increasing the Bcl-2/Bax ratio and attenuating kidney cell apoptosis. Our results indicated that continuous HRSS treatment was beneficial in preventing and treating renal I/R injury.

Hydrogen, a safe and effective antioxidant with minimal side effects, can effectively neutralize hydroxyl radicals. Several studies have demonstrated the protective effects of H_2_ by using HRSS to increase cellular antioxidative defense mechanisms. Ohsawa et al. (2007) first reported that hydrogen gas had a protective effect against cerebral I/R injury. In addition, other studies have reported that hydrogen gas can protect against organ dysfunction induced by various I/R injuries (Chen et al., 2011; Lee et al., 2015).

Renal I/R injury is common in several clinical situations, including renal transplantation and shock. I/R-induced acute renal failure is associated with decreased allograft survival in kidney transplant patients and with high mortality and morbidity in patients with native kidneys. Many studies have shown that hydrogen can inhibit the expression of inflammatory cytokines, thereby reducing renal I/R injury (Koning et al., 2015; Shi et al., 2015). However, in these studies expression of various inflammatory cytokines and apoptosis-related proteins in the kidney tissues was only measured at 24 h after I/R injury (Shingu et al., 2010; Wang et al., 2011). No detailed study on the subsequent recovery of renal function has been reported. Therefore, in this study, we compared the protective effects of long-term and short-term HRSS treatment on renal function after I/R injury and we analyzed the protective mechanisms.

Creatinine and BUN are the most commonly used markers for renal function assessment. Previous studies have found that serum Cr and BUN levels in rats treated with HRSS after renal I/R injury were significantly decreased (Shingu et al., 2010; Wang et al., 2011, 2014). However, I/R injury is a sustained injury in the body. Previous studies only reported that hydrogen had a protective effect on renal function at 24 h after I/R injury and did not study the subsequent renal recovery. Our results demonstrate that 108 h of continuous HRSS treatment promoted renal function recovery more rapidly than the short-term 24 h HRSS treatment. In addition, serum Cr and BUN in rats in the 24 h HRSS treatment group significantly increased after discontinuation of the HRSS treatment, suggesting that continuous HRSS treatment was beneficial for renal I/R injury recovery.

Several studies have demonstrated that the increase of pro-inflammatory cytokines such as IL-6 and TNF-α represents one of the first renal inflammatory responses to hyperoxia, which leads to renal tissue damage (Chen et al., 2015; Seo and Jeong, 2015). These cytokines play important roles in neutrophil activation and infiltration and induce both localized tissue injury and remote organ injury. In the present study, TNF-α and IL-6 concentrations in renal tissues were significantly increased in I/R-induced renal injury. However, HRSS treatment reversed the levels of inflammatory mediators while protecting the renal tissue against I/R-induced oxidative injury. Thus, hydrogen significantly reduced these proinflammatory cytokines in kidney tissue, suggesting that its protective effect on kidney injury might be mediated by the suppression of the intense inflammatory response and its downstream cascade. Our study has shown that continuous hydrogen administration could inhibit IL-6 and TNF-α expression and thus protect renal function.

Bcl-2 exerts anti-apoptotic effects through antioxidant activity or inhibition of ROS generation. In contrast, Bax plays an inductive role, promoting apoptosis through antagonizing Bcl-2. A previous study by Korsmeyer et al. (1993) suggested that the ratio of Bcl-2 to Bax determines whether apoptosis is induced or inhibited. In the present study, we examined the anti-apoptotic protein Bcl-2 and the pro-apoptotic protein Bax in kidney tissue. Our results demonstrated that HRSS treatment can decrease Bcl-2 and increase Bax protein expression. Our results suggested that H_2_ protective mechanism was mediated by upregulation of the Bcl-2:Bax ratio, thereby reducing kidney apoptosis induced I/R injury.

The final pathway leading to apoptosis is the activation of a series of proteases called caspases. Because the intrinsic (caspase-9) and extrinsic (caspase-8) apoptotic pathways converge at the activation of caspase-3, we measured renal caspase-3, caspase-8, and caspase-9 expressions (Juraver-Geslin and Durand, 2015; Kim et al., 2015; Monie and Bryant, 2015). Our results showed that HRSS reduced theirs expressions, suggesting that hydrogen could inhibit apoptosis through multiple pathways.

## Conclusion

The results of this study demonstrated that HRSS treatment attenuated renal I/R injury in a rat model. Taken together, our results suggested that HRSS exerted anti-apoptotic and anti-inflammatory effects to promote renal function recovery after I/R injury in rats. Although the mechanisms involved in hydrogen’s protective role remain to be fully elucidated, peritoneal injection of hydrogen could represent an easy to use, safe, cost efficient and effective novel approach for future kidney injury protection.

## Author Contributions

Conceived and designed the experiments: PY, JL, and ZH. Performed the experiments: JL and HL. Analyzed the data: JZ, LC, and SY. Contributed reagents/materials/analysis tools: XC.

## Conflict of Interest Statement

The authors declare that the research was conducted in the absence of any commercial or financial relationships that could be construed as a potential conflict of interest.

## References

[B1] Bali D. E. Osman M. A. El Maghraby G. M. (2015). Enhancement of dissolution rate and intestinal stability of clopidogrel hydrogen sulfate. *Eur. J. Drug Metab. Pharmacokinet.* 10.1007/s13318-015-0311-4 [Epub ahead of print]. 26620370

[B2] Cai J. Kang Z. Liu K. Liu W. Li R. Zhang J. H. (2009). Neuroprotective effects of hydrogen saline in neonatal hypoxia-ischemia rat model. *Brain Res.* 1256 129–137. 10.1016/j.brainres.2008.11.048 19063869

[B3] Chen H. Sun Y. P. Hu P. F. Liu W. W. Xiang H. G. Li Y. (2011). The effects of hydrogen-rich saline on the contractile and structural changes of intestine induced by ischemia-reperfusion in rats. *J. Surg. Res.* 167 316–322. 10.1016/j.jss.2009.07.045 19932899

[B4] Chen S. W. Zhu J. Zuo S. Zhang J. L. Chen Z. Y. Chen G. W. (2015). Protective effect of hydrogen sulfide on TNF-alpha and IFN-gamma-induced injury of intestinal epithelial barrier function in Caco-2 monolayers. *Inflamm. Res.* 64 789–797. 10.1007/s00011-015-0862-5 26249853

[B5] Cheng P. Chen K. Xia Y. Dai W. Wang F. Shen M. (2014). Hydrogen sulfide, a potential novel drug, attenuates concanavalin A-induced hepatitis. *Drug Des. Devel. Ther.* 8 1277–1286. 10.2147/DDDT.S66573 PMC4166909 25246769

[B6] Du H. Sheng M. Wu L. Zhang Y. Shi D. Weng Y. (2016). Hydrogen-rich saline attenuates acute kidney injury after liver transplantation via activating p53-mediated autophagy. *Transplantation* 100 563–570. 10.1097/TP.0000000000001052 26714124

[B7] Fukuda K. Asoh S. Ishikawa M. Yamamoto Y. Ohsawa I. Ohta S. (2007). Inhalation of hydrogen gas suppresses hepatic injury caused by ischemia/reperfusion through reducing oxidative stress. *Biochem. Biophys. Res. Commun.* 361 670–674. 10.1016/j.bbrc.2007.07.088 17673169

[B8] Fukui S. Ookawara T. Nawashiro H. Suzuki K. Shima K. (2002). Post-ischemic transcriptional and translational responses of EC-SOD in mouse brain and serum. *Free Radic. Biol. Med.* 32 289–298. 10.1016/S0891-5849(01)00804-8 11827754

[B9] Habib R. Begum S. Alam G. Ali A. Khan I. Waseem M. (2015). Transcription profile of genes affected in response to pathological changes in drug-induced rat model of acute kidney injury. *Ren. Fail.* 37 1225–1231. 10.3109/0886022X.2015.1057801 26114661

[B10] Han B. Zhou H. Jia G. Wang Y. Song Z. Wang G. (2016). MAPKs and Hsc70 are critical to the protective effect of molecular hydrogen during the early phase of acute pancreatitis. *FEBS J.* 283 738–756. 10.1111/febs.13629 26683671

[B11] Hayashida K. Sano M. Ohsawa I. Shinmura K. Tamaki K. Kimura K. (2008). Inhalation of hydrogen gas reduces infarct size in the rat model of myocardial ischemia-reperfusion injury. *Biochem. Biophys. Res. Commun.* 373 30–35. 10.1016/j.bbrc.2008.05.165 18541148

[B12] Juraver-Geslin H. A. Durand B. C. (2015). Early development of the neural plate: new roles for apoptosis and for one of its main effectors caspase-3. *Genesis* 53 203–224. 10.1002/dvg.22844 25619400

[B13] Katakura M. Hashimoto M. Tanabe Y. Shido O. (2012). Hydrogen-rich water inhibits glucose and alpha, beta -dicarbonyl compound-induced reactive oxygen species production in the SHR.Cg-Leprcp/NDmcr rat kidney. *Med. Gas Res.* 2:18 10.1186/2045-9912-2-18 PMC3444324 22776773

[B14] Kim B. Srivastava S. K. Kim S. H. (2015). Caspase-9 as a therapeutic target for treating cancer. *Expert Opin. Ther. Targets* 19 113–127. 10.1517/14728222.2014.961425 25256701

[B15] Koning A. M. Frenay A. R. Leuvenink H. G. Van Goor H. (2015). Hydrogen sulfide in renal physiology, disease and transplantation–the smell of renal protection. *Nitric Oxide* 46 37–49. 10.1016/j.niox.2015.01.005 25656225

[B16] Korsmeyer S. J. Shutter J. R. Veis D. J. Merry D. E. Oltvai Z. N. (1993). Bcl-2/Bax: a rheostat that regulates an anti-oxidant pathway and cell death. *Semin. Cancer Biol.* 4 327–332. 8142617

[B17] Lee D. Park S. Bae S. Jeong D. Park M. Kang C. (2015). Hydrogen peroxide-activatable antioxidant prodrug as a targeted therapeutic agent for ischemia-reperfusion injury. *Sci. Rep.* 5:16592 10.1038/srep16592 PMC4643254 26563741

[B18] Malik R. Ferguson A. V. (2015). Hydrogen sulfide depolarizes neurons in the nucleus of the solitary tract of the rat. *Brain Res.* 1633 1–9. 10.1016/j.brainres.2015.12.029 26721687

[B19] Matsushita T. Kusakabe Y. Kitamura A. Okada S. Murase K. (2011). Protective effect of hydrogen-rich water against gentamicin-induced nephrotoxicity in rats using blood oxygenation level-dependent MR imaging. *Magn. Reson. Med. Sci.* 10 169–176. 10.2463/mrms.10.169 21959999

[B20] Melcher R. L. Moerschbacher B. M. (2016). An improved microtiter plate assay to monitor the oxidative burst in monocot and dicot plant cell suspension cultures. *Plant Methods* 12:5 10.1186/s13007-016-0110-1 PMC4729151 26819624

[B21] Monie T. P. Bryant C. E. (2015). Caspase-8 functions as a key mediator of inflammation and pro-IL-1beta processing via both canonical and non-canonical pathways. *Immunol. Rev.* 265 181–193. 10.1111/imr.12284 25879293

[B22] Nagpure B. V. Bian J. S. (2016). Interaction of hydrogen sulfide with nitric oxide in the cardiovascular system. *Oxid. Med. Cell. Longev.* 2016:6904327 10.1155/2016/6904327 PMC4657111 26640616

[B23] Nakamura K. Yamagishi S. Matsui T. Yoshida T. Takenaka K. Jinnouchi Y. (2007). Pigment epithelium-derived factor inhibits neointimal hyperplasia after vascular injury by blocking NADPH oxidase-mediated reactive oxygen species generation. *Am. J. Pathol.* 170 2159–2170. 10.2353/ajpath.2007.060838 17525281 PMC1899461

[B24] Ohsawa I. Ishikawa M. Takahashi K. Watanabe M. Nishimaki K. Yamagata K. (2007). Hydrogen acts as a therapeutic antioxidant by selectively reducing cytotoxic oxygen radicals. *Nat. Med.* 13 688–694. 10.1038/nm1577 17486089

[B25] Perico N. Cattaneo D. Sayegh M. H. Remuzzi G. (2004). Delayed graft function in kidney transplantation. *Lancet* 364 1814–1827. 10.1016/S0140-6736(04)17406-0 15541456

[B26] Seo S. H. Jeong G. S. (2015). Fisetin inhibits TNF-alpha-induced inflammatory action and hydrogen peroxide-induced oxidative damage in human keratinocyte HaCaT cells through PI3K/AKT/Nrf-2-mediated heme oxygenase-1 expression. *Int. Immunopharmacol.* 29 246–253. 10.1016/j.intimp.2015.11.014 26590114

[B27] Sharples E. J. Thiemermann C. Yaqoob M. M. (2005). Mechanisms of disease: cell death in acute renal failure and emerging evidence for a protective role of erythropoietin. *Nat. Clin. Pract. Nephrol.* 1 87–97. 10.1038/ncpneph0042 16932374

[B28] Sheu S. S. Nauduri D. Anders M. W. (2006). Targeting antioxidants to mitochondria: a new therapeutic direction. *Biochim. Biophys. Acta* 1762 256–265. 10.1016/j.bbadis.2005.10.007 16352423

[B29] Shi Q. Liao K. S. Zhao K. L. Wang W. X. (2015). Hydrogen-rich saline attenuates acute renal injury in sodium taurocholate-induced severe acute pancreatitis by inhibiting ROS and NF-kappaB pathway. *Mediators Inflamm.* 2015:685043 10.1155/2015/685043 PMC4386702 25878401

[B30] Shingu C. Koga H. Hagiwara S. Matsumoto S. Goto K. Yokoi I. (2010). Hydrogen-rich saline solution attenuates renal ischemia-reperfusion injury. *J. Anesth.* 24 569–574. 10.1007/s00540-010-0942-1 20480186

[B31] Stangenberg S. Nguyen L. T. Chen H. Al-Odat I. Killingsworth M. C. Gosnell M. E. (2015). Oxidative stress, mitochondrial perturbations and fetal programming of renal disease induced by maternal smoking. *Int. J. Biochem. Cell Biol.* 64 81–90. 10.1016/j.biocel.2015.03.017 25849459

[B32] Sun L. Dutta R. K. Xie P. Kanwar Y. S. (2016). Myoinositol oxygenase over-expression accentuates generation of reactive oxygen species and exacerbates cellular injury following high glucose ambience: a new mechanism relevant to the pathogenesis of diabetic nephropathy. *J. Biol. Chem.* 291 5688–5707. 10.1074/jbc.M115.669952 26792859 PMC4786708

[B33] Wang F. Yu G. Liu S. Y. Li J. B. Wang J. F. Bo L. L. (2011). Hydrogen-rich saline protects against renal ischemia/reperfusion injury in rats. *J. Surg. Res.* 167 e339–e344. 10.1016/j.jss.2010.11.005 21392793

[B34] Wang J. F. Zha Y. F. Li H. W. Wang F. Bian Q. Lai X. L. (2014). Screening plasma miRNAs as biomarkers for renal ischemia-reperfusion injury in rats. *Med. Sci. Monit.* 20 283–289. 10.12659/MSM.889937 24553149 PMC3937038

[B35] Yemm A. Adams D. Kalia N. (2015). Targeting the delivery of systemically administered haematopoietic stem/progenitor cells to the inflamed colon using hydrogen peroxide and platelet microparticle pre-treatment strategies. *Stem Cell Res.* 15 569–580. 10.1016/j.scr.2015.10.001 26479027

